# Non-Photochemical Quenching under Drought and Fluctuating Light

**DOI:** 10.3390/ijms23095182

**Published:** 2022-05-06

**Authors:** Artur Nosalewicz, Karolina Okoń, Maria Skorupka

**Affiliations:** Institute of Agrophysics of Polish Academy of Sciences, 20-290 Lublin, Poland; k.okon@ipan.lublin.pl (K.O.); m.skorupka@ipan.lublin.pl (M.S.)

**Keywords:** *Arabidopsis thaliana*, dissipation of excessive energy, non-photochemical quenching, variable light, NPQ, RWC, PsbS, water deficit, xanthophyll cycle

## Abstract

Plants grow in a variable environment in regard to soil water and light driving photochemical reactions. Light energy exceeding plant capability to use it for photochemical reactions must be dissipated by processes of non-photochemical quenching (NPQ). The aim of the study was to evaluate the impact of various components of NPQ on the response of *Arabidopsis thaliana* to fluctuating light and water availability. A laboratory experiment with *Arabidopsis thaliana* wild type (WT) and mutants npq1 and npq4 grown under optimum or reduced water availability was conducted. Dark-adapted plants were illuminated with fluctuating light (FL) of two intensities (55 and 530 μmol m^−2^ s^−1^) with each of the phases lasting for 20 s. The impact of water availability on the role of zeaxanthin and PsbS protein in NPQ induced at FL was analysed. The water deficit affected the dynamics of NPQ induced by FL. The lack of zeaxanthin or PsbS reduced plant capability to cope with FL. The synergy of both of these components was enhanced in regard to the amplitude of NPQ in the drought conditions. PsbS was shown as a component of primary importance in suiting plant response to FL under optimum and reduced water availability.

## 1. Introduction

Light at the leaf surface often fluctuates, temporarily exceeding the plant’s capability to utilise whole light energy for photosynthesis. Changes in light intensity occur at scales in the range of seasons down to a few seconds, as lower leaves are shaded by upper ones moved by the wind. These conditions force plants to adjust the ratio between energy utilisation and dissipation. Such a response is enhanced by accompanying abiotic stresses such as drought. The combined action of variable light and drought is typical in field conditions [1].

During periods of low light, plants increase their potential to maximize the efficiency of energy absorption. The opposite situation is observed when plants exposed to high-intensity irradiance absorb excessive energy that cannot be used in photochemistry. In such conditions, reactive oxygen species (ROS) harming antenna pigments and closing reaction centres in photosystems by rapid saturation may be generated [2]. To avoid these harmful effects, several photoprotective mechanisms of thermal dissipation of excessive energy are induced to minimise damage to the photosystems. A side effect of this photoprotective response called non-photochemical quenching of chlorophyll fluorescence (NPQ) is the decline in photosynthetic efficiency. NPQ is the main and fastest response to over-excitation, consisting of several components that participate in energy dissipation differently and at different time scales. The main components of NPQ consist of [3] energy-dependent (qE), zeaxanthin-dependent (qZ), and photoinhibitory quenching (qI). qE is a rapidly reversing and slowly relaxing component of NPQ triggered by ΔpH and closely related to PsbS expression. qZ is a component associated with the xanthophyll cycle and controlled by ΔpH as well. The action of qI is related to the photoinhibition of photosystem II (PSII). The qZ component is characterized by a slower relaxation rate of 10–15 min in comparison with that of qE: 10 s–5 min [2,3].

Although NPQ helps in coping with high light (HL), frequent changes in light intensity limit plant productivity by up to 20% [2]. Quick and efficient changes in response to low light (LL) and HL are impossible due to differences in the kinetics of xanthophyll epoxydation and de-epoxydation [4]. The rate of NPQ induction is much faster than the relaxation, and the lag between the processes is exacerbated after each significant change in light intensity. The loss in plant productivity comes from the fact that the bigger the amount of dissipated energy, the lower the rate of carbon dioxide (CO_2_) assimilation, as these two processes are inversely related [5].

The PsbS protein is involved in modulating the response to water deficit conditions. It was demonstrated that it contributes to the regulation of stomatal opening, contributing to more efficient water use in water deficit conditions [6]. Xanthophylls were shown to play an important role in the mitigation of the negative impact of growth conditions through scavenging ROS generated at drought [7,8] and minimising damage to PSII.

The novelty of the study is evaluation of the importance of NPQ components in the combined action of water deficit and FL of short low and high light phases that are common in the natural environment but have not been well recognised until now. The hypothesis that processes induced by water deficit related to intensively generated ROS may appear at irradiation with FL was tested in the study. The aim of the study was to evaluate the contribution of PsbS and Zx to the process of NPQ induced by simultaneous action of FL and water deficit.

## 2. Results

The results of the preliminary experiment on the wild type of *Arabidopsis thaliana* (*At*) (Figure S1) clearly show that light fluctuations affect NPQ differently than constant light (CL) of similar average intensity. The first period of illumination with fluctuating light (0–100 s) was characterized by a similar increase in NPQ in both objects despite the periodic changes in PAR in the treatment with FL. During the following period (100–400 s), relatively high NPQ was induced by CL, compared to averaged NPQ induced by FL (NPQ_CL_ was closer to maximum than minimum momentary values of NPQ_FL_). In the later phase lasting until the end of illumination, the trends were changed and NPQ_FL_ at both LL and HL was gradually increasing. As a result, at the end of the illumination period, i.e., after c.a. 7200 s, NPQ_CL_ (280 mmol m^−2^ s^−1^ PAR) was similar to NPQ_FL_ during the LL phase (55 mmol m^−2^ s^−1^ PAR). The differences in the light conditions also affected the rate of NPQ relaxation during the dark period. NPQ relaxation in the dark recorded in the FL treatment was much faster than in CL, but this differentiation could be a result of the different light conditions at the light off (280 and 530 μmol m^−2^ s^−1^ in CL and FL, respectively).

**Figure 1:**
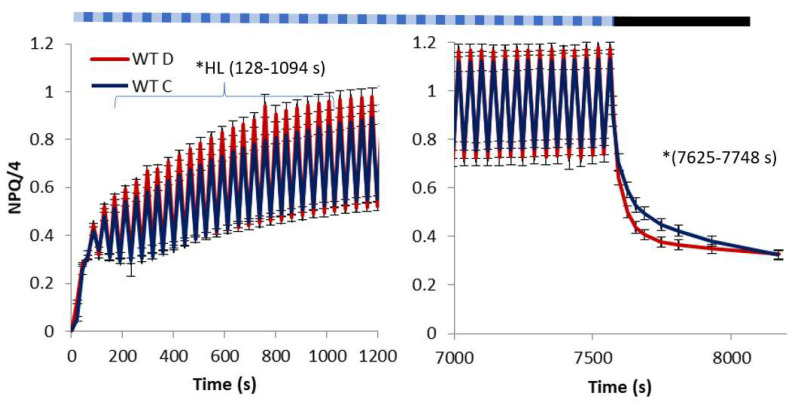
Non-photochemical quenching (NPQ) in *Arabidopsis thaliana* WT grown in well-watered (C) and water deficit (D) conditions during 7562 s of illumination with fluctuating light consisting of LL (55 μmol m^−2^ s^−1^ PAR) and HL (530 μmol m^−2^ s^−1^ PAR) phases and a following dark period. * indicates a statistical difference at *p* < 0.05 between the treatments in the HL or LL phase of fluctuating light or in the dark period. The pattern of actinic light at the top is schematic and does not reflect the real LL/HL intervals. Means and standard error of means are shown, *n* = 8.

The response of *At* WT to water deficit (Figure 1) in terms of the dissipation of NPQ was characterised by higher values of NPQ between 128 and 1094 s of illumination in the HL phase of FL than in the well-watered plants. The absence of differences in the LL phase between the treatments indicates more rapid induction and relaxation of NPQ in plants subjected to water deficit during that period. Although no significant difference was noted in the later period of illumination with FL, a more rapid decrease in NPQ occurred in the dark period shortly after actinic light (AL) turn off. However, given the trends of the NPQ decrease in the dark, stabilisation of NPQ at the higher level in the drought-treated plants could be expected.

**Figure 2:**
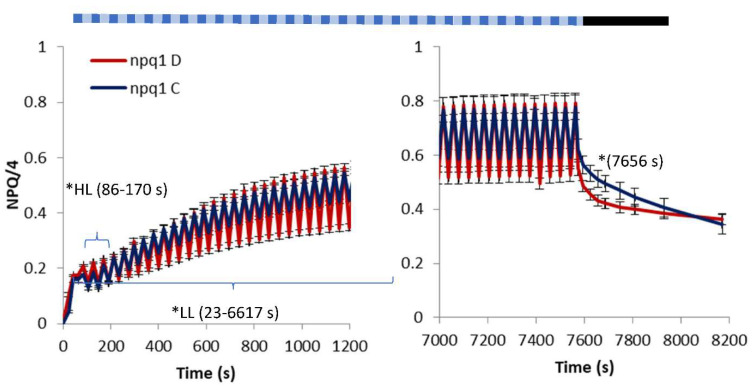
Non-photochemical quenching (NPQ) in *Arabidopsis thaliana* (npq1) grown with fluctuating light (FL) consisting of LL (55 μmol m^−2^ s^−1^ PAR) and HL (530 μmol m^−2^ s^−1^ PAR) phases and a following dark period. * indicates a statistical difference at *p* < 0.05 between the treatments in the HL or LL phase of fluctuating light or in the dark period. The indicated range 23–6617 s includes two values of NPQ for C and D treatments at 65 and 191 s that are not statistically different. The pattern of actinic light is schematic and does not reflect the real LL/HL intervals. Means and standard error of means from the initial and final periods of the experimental procedure are shown, *n* = 8.

The lack of VDE in *At* npq1 limited its ability to dissipate excessive light energy through the formation of Zx. This can be clearly seen (Figure 2) in the lower levels of NPQ compared to *At* WT (Figure 1) as in the HL and LL phases irrespective of water availability. In contrast to *At* WT, the npq1 plants varying in water availability differed in NPQ mostly in the LL phase of FL, indicating quicker NPQ relaxation at water deficit. This hypothesis could be supported by the course of NPQ relaxation in the dark, initially quicker for the D plants, being an indicator of the relaxation rate of the qE component.

**Figure 3:**
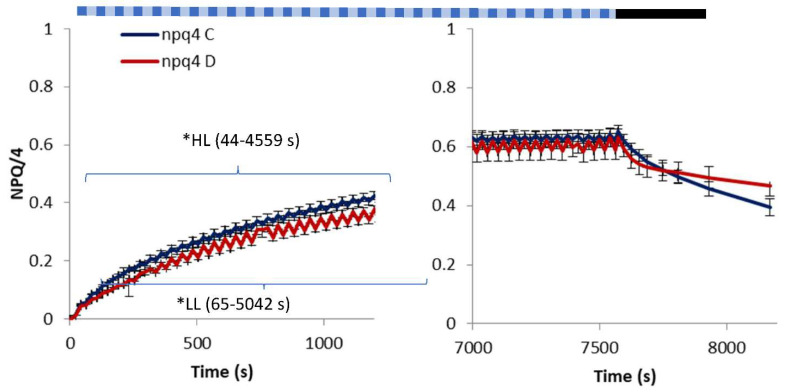
Non-photochemical quenching in *Arabidopsis thaliana* leaves (npq4) grown in well-watered (C) and water deficit (D) conditions during 7562 s of illumination with fluctuating light consisting of LL (55 μmol m^−2^ s^−1^ PAR) and HL (530 μmol m^−2^ s^−1^ PAR) phases and a following dark period. * indicates a statistical difference at *p* < 0.05 between treatments in the HL or LL phase of fluctuating light. The pattern of actinic light is schematic and does not reflect the real LL/HL intervals. Means and standard error of means from the initial and final period of the experimental procedure are shown, *n* = 8.

*Arabidopsis* npq4 responded differently to FL than the WT and npq1 plants (Figure 3). The lack of the PsbS protein generally reduced the NPQ value, and the range of changes in response to light intensity changes was diminished. The apparent low oscillations of NPQ in both of the treatments (C and D) did not differ statistically significantly for most of the AL illumination time, i.e., NPQ at HL was not significantly different from the NPQ measured subsequently at LL. The exception was the short period at the beginning of illumination, which was characterised by a significant temporal increase in NPQ irrespective of light intensity changes (for up to 128 s since the start of FL). Despite the negligible difference in the response to the LL and HL phases of FL, the NPQ in npq4 grown under water deficit reached lower values than the well-watered genotype. The pattern of the NPQ response of variously treated npq4 mutants to FL became statistically indistinguishable after the longer illumination (from 5042 s) and in the dark phase. It should, however, be noted that these NPQ changes had a stable reverse pattern compared to the light intensity and the response of WT and npq1. The 10-min period of relaxation in the dark shows that, in water deficit conditions, photoinhibition in this genotype is significantly higher (*p* < 0.005) than in both WT and npq1. Such differentiation was not observed in the control conditions.

The synergistic effect of both Zx and PsbS present in WT can be seen at the initial time of irradiation with FL (107–128 s), where the sum of NPQ in both mutants ranged from 58.9% to 71.2% of NPQ. The lowest value was noted at HL, while the highest value was observed in the LL phase of FL indicating a beneficial impact of the presence of PsbS and Zx in WT at HL. At the end of the irradiation period, the sum (npq1 + npq4) for the LL as HL phase exceeded the NPQ in WT. Such an increase in NPQ with time probably resulted from the induction of various additional components of NPQ in npq1 and npq4 and the need to dissipate excessive light energy.

**Figure 4:**
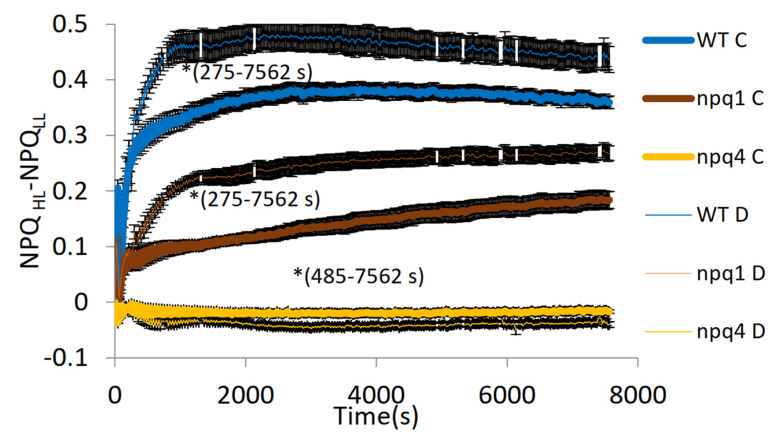
Amplitude of non-photochemical quenching calculated by subtraction of NPQ at HL (NPQ_HL_) from NPQ at the following LL phase (NPQ_LL_) in *Arabidopsis thaliana* WT, npq1, and npq4 in well-watered (C) and water deficit (D) conditions during illumination with fluctuating light consisting of LL (55 μmol m^−2^ s^−1^ PAR) and HL (530 μmol m^−2^ s^−1^ PAR) phases (the dark period is not shown). The negative values for npq4 result from the specific response to FL. The pattern of actinic light at the top is schematic and does not reflect the real LL/HL intervals. Means and standard error of means are shown, *n* = 8, * indicates a statistical difference at *p* < 0.05 between the C and D treatments.

Another important aspect of the response to FL is the amplitude of NPQ to the HL and LL phases. The analysis of the data shown in Figure 1, Figure 2 and Figure 3 indicates strong differentiation of the NPQ amplitude by water availability (Figure 4). The highest amplitude was noted in WT at D, reaching a maximum value of 0.48 after c.a. 35 min from the start of FL. In the C treatment, the same genotype also exhibited a trend with a maximum, but it was reached later, i.e., after c.a. 60 min. from the start of FL. The NPQ amplitude in npq1 was characterised by a constant increase throughout the FL irradiation period with a more rapid increase noted for plants in the drought variant. As mentioned earlier, npq4 exhibited a reverse response to light intensity with low amplitude, lower in C than in the D treatment, and the relationship was maintained in almost the whole irradiation period. The analyses of the quantum yields of regulated Y(NPQ) and non-regulated Y(NO) energy dissipation (Table 1) allowed us, at least partly, to explain the response of the mutant. The higher NPQ at LL than HL in npq4 results from the high values of non-regulated energy dissipation Y(NO) accounting for more than 40% of Y(NPQ). The values of the regulated component Y(NPQ) follow light intensity changes, i.e., Y(NPQ) at LL is lower than Y(NPQ) at HL.

**Figure 5:**
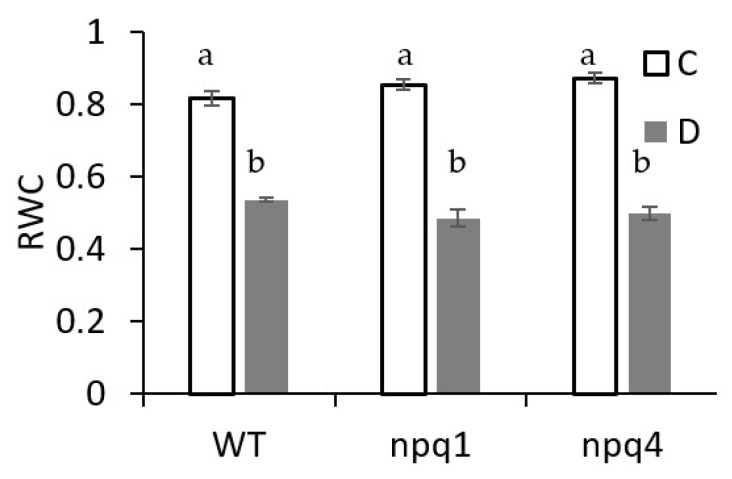
Leaf relative water content (RWC) in *Arabidopsis thaliana* WT, npq1, and npq4 plants in well-watered (C) and drought (D) conditions maintained for 16 days. Means and standard errors are shown, *n* = 8.

The values of leaf relative water content RWC (Figure 5) measured immediately after irradiation with FL show that the level of water deficit in the pots lowered RWC significantly and its value did not differ among the plant genotypes subjected to the same treatment. The leaf RWC level in the water deficit conditions had the highest value in WT (54%) but declined down to 50% and 48% in npq4 and npq1, respectively.

## 3. Discussion

Variable light and drought are common in the natural environment, and the overall spectrum of their combined impact by far exceeds their single action. Non-detrimental light intensity in optimum conditions may become excessive when accompanied by drought. Despite the high differentiation in the action of FL and water deficit, plant response to their stressful level is characterised by specified similarity, e.g., ROS generation at exposure to both stressors, involvement of xantophylls in abscisic acid biosynthesis, and ROS scavenging. Such interrelationships may aggravate or diminish the impact of these environmental factors on vegetation. Schwarz et al. (2015) [9] demonstrated that zeaxanthin epoxidase, i.e., an important enzyme for acclimation to light and drought stresses is degraded in leaves in water deficit conditions.

It has also been suggested [10] that the response to dehydration predominates over the effect of light on the expression of genes regulating the xanthophyll cycle; however, this relationship may vary depending on the intensity of the stressors. The complexity of plant response is enhanced when the level of the stressor is changing with time. Functioning of the photosynthetic apparatus under dynamically changing irradiation differs from steady state conditions [5], which can be clearly seen in the comparison of the trends of NPQ induced by constant or variable light (Figure S1).

The relaxation kinetics of NPQ is among the characteristics playing an important role in the switch from HL to LL [5]. Quick and efficient quenching of excitation prevents the generation of toxic ROS at FL. Conversion of violaxanthin (Vx) via antheraxanthin (Ax) to Zx at 25 °C takes in total 8.6 min, and such conversion is deregulated under FL [11]. Due to the much slower reverse process of de-epoxidation of Zx, the accumulation of Zx occurs in the conditions of FL, compared to CL of similar intensity (Figure S1). As reported by Kress and Jahns (2017) [12], the increase in Zx is slower than NPQ induction in *At* WT but similar rates of Zx and NPQ induction are noted in npq4. Moreover, their study shows independence of NPQ of the zeaxanthin level.

Due to its variable nature highlighted in our experimental conditions, AL induces various mechanisms contributing to NPQ with specific time scales. Such conditions allowed the observance of a phenomenon called “light memory” [12,13]. A crucial role in the mechanism is played by Zx. The mechanism in NPQ is reflected in the extent of quenching. It depends on the duration and intensity of previous exposure to AL and is evident in the secondary exposure to AL. The rapidly protonated antennae PsbS-H enhance quenching when Zx accumulated at previous exposure to AL is still present. Such a response could be responsible for the improved NPQ level and amplitude observed in *At* W (Figure 1 and Figure 4), compared to the other genotypes. Welc et al. (2021) [14] suggested that PsbS allows the exchange of xanthophylls in thylakoid membranes, thus contributing to the synergistic effect of PsbS and Zx. However, in the conditions of gradually increasing light intensity in their experiment, the synergistic effect of Zx and PsbS was substantially higher than the effect observed in our experiment under FL.

The changes in NPQ (Figure 2) show, despite the lack of Zx in npq1, a relatively rapid increase in NPQ, which can be attributed to the presence of the PsbS protein and lutein [15]. As shown by Li et al. (2009) [16], npq1 contains a similar amount of lutein to that in WT plants. Lutein was observed to contribute directly to qE, which is a rapid NPQ component, by substituting Zx in quenching the excitation from chlorophyll [16]. As shown by Demmig-Adams et al. (2020b) [17], the lutein level remains constant in contrast to the variable content of Zx in leaves in response to light intensity, which explains the reduced amplitude of NPQ under FL (Figure 4).

Our results showing the substantial increase in NPQ in the HL phase and the accompanying water deficit in WT (Figure 1), as well as the lack of such a response in npq1 (Figure 2), could be explained by the accumulation of Zx in drought conditions. Such a response was shown in many studies [18,19,20]. It is generally related to excessive light energy under drought stress induced by stomatal closure. Increased leaf Zx in drought conditions was explained by an increased rate of degradation of zeaxanthin epoxidase catalysing the conversion of Zx to Vx [9]. Moreover, Baraldi et al. (2008) [21] observed prolonged retention of Zx and Ax during the dark period in plants exposed to drought. Retention of these xanthophylls was attributed to the enhanced photoprotection capacity of drought-stressed plants in the morning light. Peguero-Pina et al. (2008) [22] showed an increase in Ax and Zx in leaves of Kermes oak with progressive drought at the morning increase in irradiation as one of the protective mechanisms against drought-induced ROS, as suggested by Latowski et al. (2011) [7]. The quicker relaxation of NPQ in npq1 in the water deficit conditions (Figure 2) may have resulted from the increase in the level of PsbS. A nearly 30% increase in PsbS under a 15-day water deficit was reported [23] in leaves of *At* WT. A decreased NPQ relaxation time accompanying a decrease in light intensity was also shown in mutants overexpressing PsbS or plants with increased PsbS due to exposure to HL [24]. The NPQ response of *At* mutant L17 overexpressing PsbS was shown to be exceptionally responsive to rapid pulses of high light and periods of the dark [25] demonstrating an important role of PsbS in both induction and relaxation of quenching. On the other hand, the lack of similar quicker relaxation in *At* WT at water deficit could be explained by the accumulation of Zx in the WT (absent in npq1). The presence of Zx in WT was shown to decrease the kinetics of NPQ relaxation compared to Vx (present in npq1) in an experiment on intact spinach chloroplasts [26] and in *At* WT [13].

Although PsbS allows a quick NPQ response to HL, it was shown that qE can form without PsbS when ∆pH is high enough [27]. The slower rate of NPQ induction in npq4 at FL (Figure 3) with the weak response to the changes in light intensity can be attributed to the presence of only slow NPQ components. An experiment on the same plant material [14] showed that npq4 accumulated more than twice as much Zx at HL (500 μmol m^−2^ s^−1^) than WT to cope with excessive light energy. The results reported in [4] showed that npq4 at 1000 μmol m^−2^ s^−1^ of AL showed no rapid component of NPQ but displayed nearly 25% of slow and reversible NPQ observed in WT. In the conditions of FL, the response of npq4 (Figure 3) displayed an overall increase in NPQ, however, with opposite changes in NPQ to those in light intensity (Figure 4). This indicates that this mutant fails in adjusting NPQ to rapidly changing conditions. The more responsive npq1 mutant confirms the importance of PsbS in the qE component of NPQ [3]. The visible slow induction of NPQ in npq4 was explained by Johnson and Ruban (2011) [27] by induction of qE without PsbS at sufficiently high ∆pH. The specific reverse response of npq4 in terms of the NPQ values observed in our experiment (Figure 3 and Figure 4) and in a study [28] indicates the importance of PsbS in response to both an increase and a decrease in light intensity, and shows the tight relationship between the different NPQ components having different characteristic reaction times. However, the analysis of the components of non-photochemical quenching (Table 1) shows that light-regulated non-photochemical quenching Y(NPQ) follows the FL light changes. It indicates that the action of the weak active photo protective mechanisms to rapidly changing FL in npq4 is masked by harmful non-regulated quenching Y(NO). A high level of non-regulated dissipation is an indicator of conditions in which triplet state chlorophylls and ROS may be excessively formed [29].

The presence of Vx and PsbS in npq1 may be responsible for the specific response at FL. The measurements of Chl *a* fluorescence lifetimes revealed different effects of the combined action of PsbS and xanthophylls depending on light intensity [14]. Analyses of chlorophyll fluorescence lifetimes revealed a photoprotective role of Zx at HL (700 μmol m^−2^ s^−1^), while the presence of Vx at LL (100 μmol m^−2^ s^−1^) enhanced the efficiency of photosynthesis. An experiment on rice grown in moderate or HL conditions showed that both Zx and PsbS are involved in photoprotective response at moderate light, while only Zx is involved at high light [30].

Overexpression of PsbS is suggested as a tool to obtain abiotic stress-resistant crops through increased resistance to photoinhibition [25]. The importance of PsbS was indicated by the rate of relaxation of NPQ in the dark (Figure 3) in the npq4 mutant without the presence of PsbS. The significantly higher NPQ in the npq4 genotype after 10 min of relaxation in the dark is an indicator of increased photo damage [3], compared to both WT and npq1 (Figure 1 and Figure 2). Insufficient photoprotective action of xanthophyll in preventing photooxidative damage was also shown in npq4 [31].

The leaf RWC level in water deficit conditions (Figure 5) is an indicator of severe stress. However, it has to be pointed out that *Arabidopsis thaliana* is able to maintain the functioning of chloroplastic membranes, which are important for photosynthetic activity even in severe water deficit conditions through adjusting the lipid composition for a stable bilayer conformation [32]. The decrease in RWC at the regulated water deficit in our experiment is similar to withholding of watering for 12 days in *Arabidopsis thaliana* WT [23], which was associated with c.a. 32% decrease in chlorophyll content. *At* genotypes characterised by similar ABA accumulation at drought had reduced differentiation in leaf relative water content under severe water stress [33] that was similar to the response noted in our experiment.

## 4. Materials and Methods

### 4.1. Arabidopsis Thaliana

Wild type Col-0 (*At* WT) and single mutants: npq1 deficient in violaxanthin de-epoxidase (VDE) and npq4 deficient in PsbS protein (Nottingham Arabidopsis Stock Centre) were grown in plastic pots (5 × 5 × 5 cm) filled with commercial growth medium Compo Sana (, COMPO, Münster, Germany) in growth chambers KK1200 (, Pol-Eko, Wodzisław Śląski, Poland). The plants were grown at a temperature of 22 °C during 8-h day and 18 °C during the night, light intensity of photosynthetically active radiation of 130 μmol m^−2^ s^−1^, and 60% relative air humidity. The plants were watered to field capacity with half strength Hoagland solution every 2 days. Starting from the 30th day from sowing, the watering doses were adjusted to maintain:

- pot capacity for plants grown at optimum soil water availability (C);
- 60% of pot capacity for plants grown in water deficit conditions (D);

The term pot capacity corresponds to field capacity applied in laboratory conditions for soil material packed into pots [34].

### 4.2. Determination of Chlorophyll Fluorescence

The response of the plants to FL and water deficit was measured in 60- to 75-day-old plants. The measurements were conducted using Imaging PAM Maxi equipped with an IMAG-K7 CCD camera (Walz, Effeltrich, Germany). Three individuals: WT, npq1, and npq4 were placed together in the chamber of IMAGING PAM during each single measurement. Prior to the measurements, the plants were dark adapted for 30 min then illuminated with:

- light of constant intensity equal to 280 μmol m^−2^ s^−1^ for 7562 s followed by a 560-s period of relaxation in the dark;
- fluctuating light characterized by frequent (20 s long) changes from low (55 μmol m^−2^ s^−1^) to high intensity (530 μmol m^−2^ s^−1^) lasting in total for 7562 s followed by a 560-s period of relaxation in the dark.

Blue light (450 nm) was used as pulse-modulated measuring light (ML), actinic illumination, and for saturation pulses. All levels of AL include the intensity of ML. The air temperature and humidity were the same as during the day conditions in the growth chamber.

### 4.3. Measurements of Leaf RWC

Immediately after measurements of fluorescence, the leaves of irradiated plants were detached to determine relative water content (RWC). The leaves were weighed (FW) and submerged in deionised water for 24 h in the dark, weighed again (TW), and placed for another 24 h in a dryer set at a temperature of 75 °C to measure dry weight (DW). The results were substituted into the formula

$$\mathrm{RWC}=\frac{\mathrm{FW}-\mathrm{DW}}{\mathrm{TW}-\mathrm{DW}}$$

### 4.4. Statistical Analysis

All measurements were conducted in 4 repetitions and the experiment was repeated twice. The results were analysed by ANOVA (Tibko, Statistica 13.3). The means were compared with the use of the post-hoc HSD Tukey test at *p* < 0.05.

To ensure clarity, all records of the experimental data of NPQ shown in the figures were divided into two parts: the initial phase (0–1200 s) and the last part (7000–8200 s) including the dark period. The middle part was not displayed, as the data during this period can be estimated from trends visible in both figures.

## 5. Conclusions

The illumination of Arabidopsis thaliana WT and its npq1 and npq4 mutants grown in water deficit conditions resulted in different patterns of the NPQ response to fluctuating light, compared to plants grown at optimum water availability. A synergistic effect of Zx and the PsbS protein on the NPQ level was displayed in the *At* WT plants affected by drought at early irradiation with FL. The drought clearly affected the response of the WT and npq1 genotypes of *At* to the low and high light phases in terms of the NPQ amplitude, which is an estimate of the fitness of plants to a variable environment.

Our results show a crucial role of PsbS in shaping the proper response to rapid light fluctuations, especially at water deficit. Our findings combined with the current knowledge on the positive impact of PsbS on plant-water relationships at drought indicate huge protective potential of the PsbS protein in conditions of a variable and unfavourable environment.

Further studies are needed to reveal the impact of FL and water deficit in cereals. It seems that for practical application, it is important to study the response of the whole plant to FL, taking into account lower leaves, which are the main plant part subjected to rapid FL, and evaluate the FL impact on the acceleration of aging, which results in loss of productivity.

## Figures and Tables

**Table 1:** Quantum yields of photochemical energy conversion (YII), light-regulated non-photochemical energy dissipation Y(NPQ) and non-regulated non-photochemical energy dissipation in PS II Y(NO) measured in the last periods of low and high light (7520–7562 s) in *Arabidopsis thaliana* WT, npq1, and npq4 in well-watered (C) and water deficit (D) conditions during illumination with fluctuating light consisting of low (LL) and high (HL) light phases.

		Y(II)		Y(NPQ)		Y(NO)	
		LL	HL	LL	HL	LL	HL
WT	C	0.417 *	0.165 **	0.439 ns	0.671 **	0.144 *	0.165 **
	D	0.356 *	0.074 **	0.481 ns	0.729 **	0.164 *	0.197 **
npq1	C	0.412 **	0.158	0.412 ns	0.633 *	0.176 **	0.210 **
	D	0.355 **	0.084	0.436 ns	0.690 *	0.210 **	0.226 **
npq4	C	0.399 **	0.175	0.432 *	0.588 ns	0.170 **	0.237 **
	D	0.303 **	0.103	0.499 *	0.625 ns	0.199 **	0.273 **

Average values for *n* = 8 are shown. * indicates significant differences between treatments differing in water availability at *p* < 0.05, ** at 0.01, ns—not significant differences at *p* < 0.05.

## Data Availability

The data presented in this study are available on request from the corresponding author.

## Supplementary Materials

The following supporting information can be downloaded at: https://www.mdpi.com/article/10.3390/ijms23095182/s1.

## Abbreviations

*At*	*Arabidopsis thaliana*
FL	fluctuating light
HL	high light
LL	low light
NPQ	non-photochemical quenching
npq1	*Arabidopsis thaliana* mutant, see description in Materials and methods
npq4	*Arabidopsis thaliana* mutant, see description in Materials and methods
PsbS	photoprotective protein from the light harvesting complex family
WT	wild type
Zx	zeaxanthin
Ax	antheraxanthin
Vx	violaxanthin
VDE	violaxanthin de-epoxidase
