# Supplementary material for: Non-Photochemical Quenching under Drought and Fluctuating Light
Source: Int J Mol Sci. 2022 May 6;23(9):5182. doi: 10.3390/ijms23095182 (PMC9105319; doi:10.3390/ijms23095182)
Supplement: Supplementary file 1 [file ijms-23-05182-s001.zip › ijms-1686626-supplementary.pdf]

International Journal of
Molecular Sciences

MDPI

Supplementary Materials:

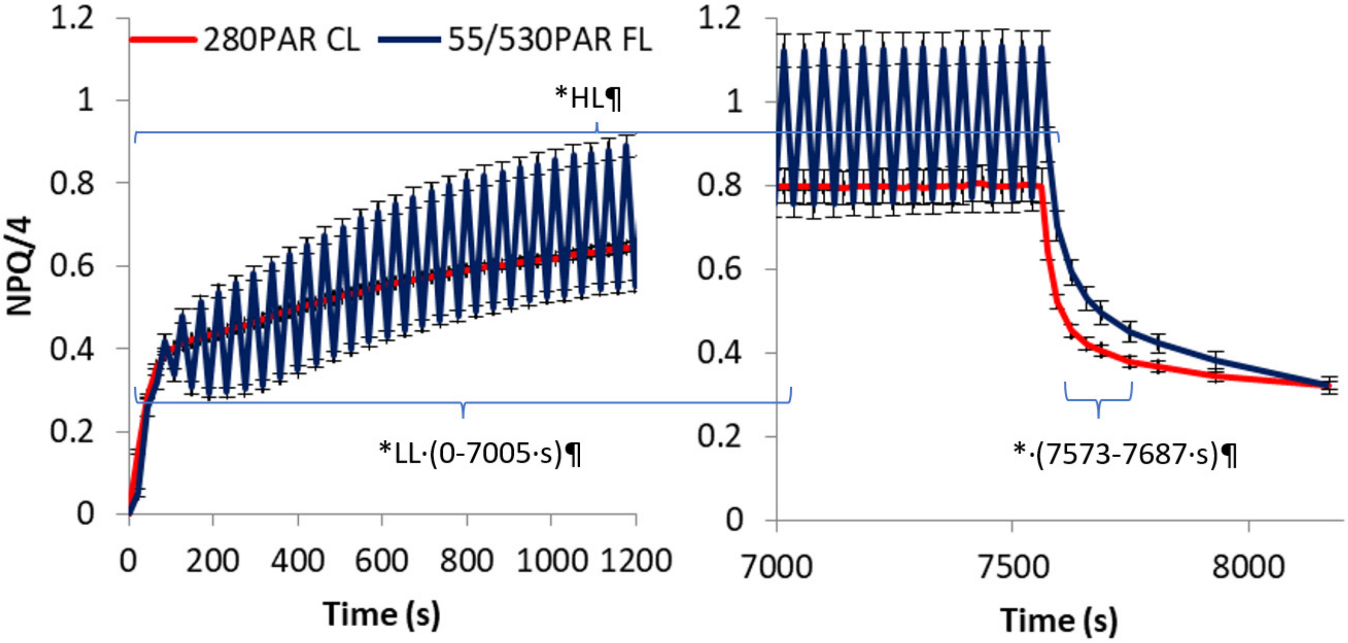

Figure S1. Non-photochemical quenching (NPQ)induced by 280 µmol $m^{-2}s^{-1}$ of constant light (280PAR CL) and fluctuating light (50/530 PAR FL) changing every 20 s from low (55 µmol $m^{-2}s^{-1}$) to high (530 µmol $m^{-2}s^{-1}$) intensity for 7562 s followed by a dark period. Data for the *At* WT grown in soil with optimum water availability. Means and standard errors are given (n=8), * indicates a statistical difference at $p < 0.05$ between the HL or LL phase of fluctuating and constant light.
